# Supplementary material for: Uncovering the hierarchical structure of self-reported hostility
Source: PLoS One. 2020 Sep 29;15(9):e0239631. doi: 10.1371/journal.pone.0239631 (PMC7523964; doi:10.1371/journal.pone.0239631)
Supplement: S1 Table — (DOCX) [file pone.0239631.s003.docx]

**S1 Table. FOA items that were removed due to extreme normality violation.**

| **Item** |
| --- |
| Threaten someone physically |
| Throw something at someone |
| Hit someone in the face or head |
| Beat someone up |
| Hold someone on the ground |
| Hurt someone physically |
| Steal someone’s things |
| Start a fire that causes damage |
| Harm someone’s property |
| Vandalize someone’s house or things |
| Damage someone’s property |
| Mess up someone’s work |
| Tease someone |
| Ruin someone’s friendship with other people |
| Isolate someone |
| Become friends with someone else as revenge |
