# Supplementary figures and images for: Uncovering the hierarchical structure of self-reported hostility
Source: PLoS One. 2020 Sep 29;15(9):e0239631. doi: 10.1371/journal.pone.0239631 (PMC7523964; doi:10.1371/journal.pone.0239631)

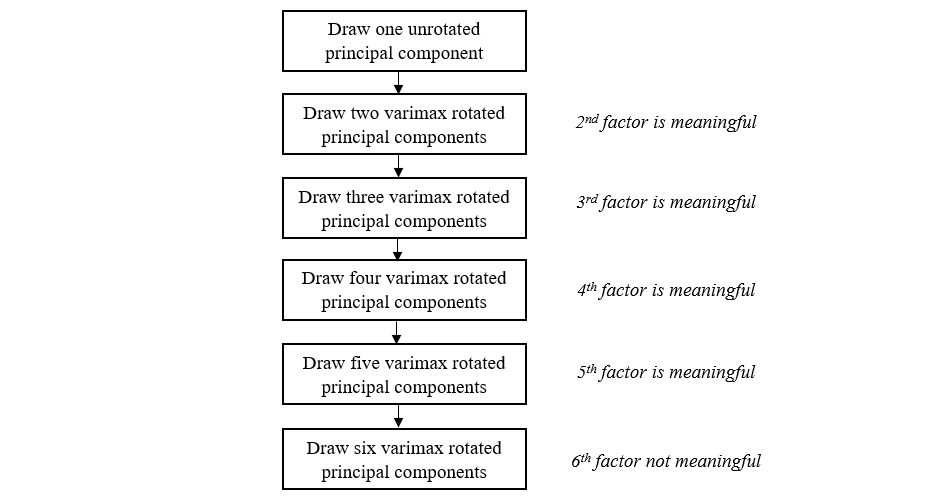

Supplement: S1 Fig — (TIF) [file pone.0239631.s005.tif]
