# Supplementary material for: Uncovering the hierarchical structure of self-reported hostility
Source: PLoS One. 2020 Sep 29;15(9):e0239631. doi: 10.1371/journal.pone.0239631 (PMC7523964; doi:10.1371/journal.pone.0239631)
Supplement: Attachment — Submitted filename: PONE-D-20-17138_reviewer.pdf [file pone.0239631.s006.pdf]

PLOS ONE

Uncovering the hierarchical structure of self-reported hostility

--Manuscript Draft--

**Manuscript Number:**	PONE-D-20-17138
**Article Type:**	Research Article
**Full Title:**	Uncovering the hierarchical structure of self-reported hostility
**Short Title:**	HIERARCHICAL STRUCTURE OF HOSTILITY
**Corresponding Author:**	Martijn Willem van Teffelen, MSc<br>Maastricht University<br>Maastricht, Limburg NETHERLANDS
**Keywords:**	Hostility; anger; aggression; psychometric properties
**Abstract:**	Hostility and other related terms like anger and aggression are often used interchangeably to describe antagonistic affect, cognition and behavior. Psychometric studies suggest that hostility consists of multiple separate factors, but consensus is currently lacking. In the present study we examined the hierarchical structure of hostility. The hierarchical structure of hostility was examined in N = 376 people (i.e., a mixed community and highly-hostile sample), using both specific and broad hostility self-report measures. A series of Principal Components Analyses revealed the structure of hostility at five levels of specificity. At intermediate levels, hostility can consistently be expressed in affective, cognitive and behavioral components. At the most specific level, hostility can be expressed in terms of Angry Affect; Hostile Intent; and Verbal, Relational, and Physical Aggression. The pattern of associations showed significant convergence, and some divergence with broad and more specific hostility measures. The present findings stress the need for novel instruments that capture each hostility facet separately to reduce conceptual confounding.
**Order of Authors:**	Martijn W van Teffelen, MSc
	Jill Lobbestael
	Marisol J. Voncken
	Frenk Peeters
**Additional Information:**	
**Question**	**Response**
**Financial Disclosure**<br><br>Enter a financial disclosure statement that describes the sources of funding for the work included in this submission. Review the submission guidelines for detailed requirements. View published research articles from *PLOS ONE* for specific examples.<br><br>This statement is required for submission and **will appear in the published article** if the submission is accepted. Please make sure it is accurate.	The author(s) received no specific funding for this work.

Powered by Editorial Manager® and ProduXion Manager® from Aries Systems Corporation

**Unfunded studies**<br>Enter: *The author(s) received no specific funding for this work.*<br><br>**Funded studies**<br>Enter a statement with the following details:<br>• Initials of the authors who received each award<br>• Grant numbers awarded to each author<br>• The full name of each funder<br>• URL of each funder website<br>• Did the sponsors or funders play any role in the study design, data collection and analysis, decision to publish, or preparation of the manuscript?<br>• **NO** - Include this sentence at the end of your statement: *The funders had no role in study design, data collection and analysis, decision to publish, or preparation of the manuscript.*<br>• **YES** - Specify the role(s) played.<br><br>* typeset	
**Competing Interests**<br><br>Use the instructions below to enter a competing interest statement for this submission. On behalf of all authors, disclose any competing interests that could be perceived to bias this work—acknowledging all financial support and any other relevant financial or non-financial competing interests.<br><br>This statement **will appear in the published article** if the submission is accepted. Please make sure it is accurate. View published research articles from *PLOS ONE* for specific examples.	The authors have declared that no competing interests exist.

Powered by Editorial Manager® and ProduXion Manager® from Aries Systems Corporation

**NO authors have competing interests**<br><br>Enter: *The authors have declared that no competing interests exist.*<br><br>**Authors with competing interests**<br><br>Enter competing interest details beginning with this statement:<br><br>*I have read the journal's policy and the authors of this manuscript have the following competing interests: [insert competing interests here]*<br><br>* typeset	
**Ethics Statement**<br><br>Enter an ethics statement for this submission. This statement is required if the study involved:<br><br>• Human participants<br>• Human specimens or tissue<br>• Vertebrate animals or cephalopods<br>• Vertebrate embryos or tissues<br>• Field research<br><br>Write "N/A" if the submission does not require an ethics statement.<br><br>General guidance is provided below. Consult the submission guidelines for detailed instructions. **Make sure that all information entered here is included in the Methods section of the manuscript.**	The Ethical Review Committee Psychology and Neuroscience at Maastricht University provided ethical approval to carry out the study (ERCPN- 167_08_05_2016). Written consent was obtained.

Powered by Editorial Manager® and ProduXion Manager® from Aries Systems Corporation

**Format for specific study types**<br><br>**Human Subject Research (involving human participants and/or tissue)**<br>• Give the name of the institutional review board or ethics committee that approved the study<br>• Include the approval number and/or a statement indicating approval of this research<br>• Indicate the form of consent obtained (written/oral) or the reason that consent was not obtained (e.g. the data were analyzed anonymously)<br><br>**Animal Research (involving vertebrate animals, embryos or tissues)**<br>• Provide the name of the Institutional Animal Care and Use Committee (IACUC) or other relevant ethics board that reviewed the study protocol, and indicate whether they approved this research or granted a formal waiver of ethical approval<br>• Include an approval number if one was obtained<br>• If the study involved *non-human primates*, add *additional details* about animal welfare and steps taken to ameliorate suffering<br>• If anesthesia, euthanasia, or any kind of animal sacrifice is part of the study, include briefly which substances and/or methods were applied<br><br>**Field Research**<br><br>Include the following details if this study involves the collection of plant, animal, or other materials from a natural setting:<br>• Field permit number<br>• Name of the institution or relevant body that granted permission	
**Data Availability**<br><br>Authors are required to make all data underlying the findings described fully available, without restriction, and from the time of publication. PLOS allows rare exceptions to address legal and ethical concerns. See the PLOS Data Policy and FAQ for detailed information.	No - some restrictions will apply

A Data Availability Statement describing where the data can be found is required at submission. Your answers to this question constitute the Data Availability Statement and **will be published in the article**, if accepted.<br><br>**Important:** Stating 'data available on request from the author' is not sufficient. If your data are only available upon request, select 'No' for the first question and explain your exceptional situation in the text box.<br><br>Do the authors confirm that all data underlying the findings described in their manuscript are fully available without restriction?	
**Describe where the data may be found in full sentences. If you are copying our sample text, replace any instances of XXX with the appropriate details.**<br><br>• If the data are **held or will be held in a public repository**, include URLs, accession numbers or DOIs. If this information will only be available after acceptance, indicate this by ticking the box below. For example: *All XXX files are available from the XXX database (accession number(s) XXX, XXX.).*<br>• If the data are all contained **within the manuscript and/or Supporting Information files**, enter the following: *All relevant data are within the manuscript and its Supporting Information files.*<br>• If neither of these applies but you are able to provide **details of access elsewhere**, with or without limitations, please do so. For example:<br><br>*Data cannot be shared publicly because of [XXX]. Data are available from the XXX Institutional Data Access / Ethics Committee (contact via XXX) for researchers who meet the criteria for access to confidential data.*<br><br>*The data underlying the results presented in the study are available from (include the name of the third party*	Data cannot be shared publicly because the data is stored at our University data respository. Data are available from the Maastricht Unviersity Institutional Data Access (contact via martijn.vanteffelen@maastrichtuniversity.nl) for researchers who meet the criteria for access to confidential data.

and contact information or URL).<br>• This text is appropriate if the data are owned by a third party and authors do not have permission to share the data.<br><br>* typeset	
Additional data availability information:	Tick here if the URLs/accession numbers/DOIs will be available only after acceptance of the manuscript for publication so that we can ensure their inclusion before publication.

Click here to download Manuscript Manuscript.docx

Uncovering the hierarchical structure of self-reported hostility

Martijn W. van Teffelen*, Jill Lobbestael, Marisol J. Voncken, Frenk Peeters

Department of Clinical Psychological Science, Maastricht University

*Corresponding author

Adress: Maastricht University, P.O. box 616, 6200 MD, Maastricht, the Netherlands.

E-mail: martijn.vanteffelen@maastrichtuniversity.nl.

Phone number: +31433882487.

This affirmation is not based on specific AHA (aggression, hostility, and anger) literature. Aggression is the behavioral, hostility the cognitive, and anger is the emotional component of the triad. Often???

Abstract

Hostility and other related terms like anger and aggression are often used interchangeably to describe antagonistic affect, cognition and behavior. Psychometric studies suggest that hostility consists of multiple separate factors, but consensus is currently lacking. In the present study we examined the hierarchical structure of hostility. The hierarchical structure of hostility was examined in N = 376 people (i.e., a mixed community and highly-hostile sample), using both specific and broad hostility self-report measures. A series of Principal Components Analyses revealed the structure of hostility at five levels of specificity. At intermediate levels, hostility can consistently be expressed in affective, cognitive and behavioral components. At the most specific level, hostility can be expressed in terms of Angry Affect; Hostile Intent; and Verbal, Relational, and Physical Aggression. The pattern of associations showed significant convergence, and some divergence with broad and more specific hostility measures. The present findings stress the need for novel instruments that capture each hostility facet separately to reduce conceptual confounding.

Provide some examples of such affirmation

Introduction

In psychological research, human antagonistic behavior and its' related cognitive-affective experiences are often operationalized by the terms anger, hostility, or aggression. Unfortunately, these and other related terms (e.g., irritability, agitation and frustration) are often used interchangeably. Some use the same term for different constructs (i.e., the jingle fallacy), while others use different terms for the same construct (i.e., the jangle fallacy) [1]. Many self-report measures in the field also use a wide array of terms that even combine two concepts such as 'anger expression', 'hostile aggression', 'affective aggression' and 'angry hostility', adding further confusion.

Adding to this confusion, two theoretical perspectives can be distinguished that conceptualize hostility and its related cognitive-affective experiences. The first, a *unidimensional perspective* conceptualizes hostility as one construct that includes the interrelated elements of cynical beliefs about others and the world, hostile attribution bias (i.e., the tendency to interpret emotionally ambiguous scenario's as hostile), angry emotional states, and aggressive behavior [2-4]. The second, a *multidimensional perspective* conceptualizes hostility in terms of a broad conceptual domain that consists out of two or more lower-level facets [5-7]. Empirical evidence tends to converge with this multidimensional perspective of hostility. That is, exploratory factor analytic studies generally find multifactorial solutions [8-11]. Typically, these studies include multiple measurements of hostility and apply exploratory factor analysis to identify the optimal number of factors. Part of the confusion surrounding the concept of hostility can be attributed to diverging results from these exploratory studies. Some previous studies demonstrated two factors such as anger expression and anger experience [9, 10, 12, 13]. Others reported three-factor solutions, distinguishing affect, behavior and cognition [9, 14, 15] – also referred to as the ABC-

Please, including here a specific citation for this affirmation

model [16], or similarly the AHA-model (i.e., anger, hostility, aggression) [5]. Finally, also a four factor model has been reported with distinctions between hostility, anger, verbal aggression, and physical aggression [8, 11]. Apparently, the findings of methodologically heterogeneous factor analytic studies hardly converge in terms of number of produced factors and factor content.

This premise requires a further explanation of why they have considered to include these (yellow tracked) expressions

I'd like to see at least a citation of this previous findings

A major caveat in the available evidence is that previous work focused on two levels of analysis: a higher order domain, trait or latent construct (e.g., hostility) and lower-level facets (e.g., experience and expression). Consequently, the outcomes of exploratory factor analyses are likely to be a function of the combination of instruments, subscales and items that were fed into the respective models. However, studies in the broad personality psychology field suggest that there is value in investigating model solutions that include more than two conceptual, hierarchical layers [17]. For example, the highly influential Big Five personality model shows that seemingly diverging results of factor analytic studies (i.e., showing different 'optimal' factor solutions) converge into a seven-layered hierarchical model in which lower-order facets become more and more specific with each hierarchical layer [18]. Other examples of presumed diverging models that converge into a multi-layered hierarchical model have been reported for intelligence or 'g' [19] and psychopathology or 'p' [20]. Along the same lines, hostility could pote[illegible] expressed as a hierarchical structure consisting of one higher order domain that clusters into two to many facets that become more specific in each additional hierarchical layer. To the best of our knowledge, no hierarchical cluster analysis on hostility has been previously performed.

g, third stratum of CHC cognitive ability model should be represented italicized

In sum, factor-analytic evidence tends to converge with a multidimensional view of the hostility construct, but previous work shows differences in number and content of factor solutions. The current study, including facet-level and broad-domain measures, therefore builds

I could not see the parallelism between hostility and personality or intelligence. The complexity of personality or intelligence are above of the hostility one. Are you considering here a psychometric approach for hostility?

on earlier work by examining the hierarchical structure of the hostility concept. The main expectation is that a multidimensional hierarchical structure will be uncovered.

Materials and methods

Participants

Participants were sampled in two ways. First, participants were recruited from the general population in Maastricht, the Netherlands through advertisement. Second, ensuring a representative distribution with enough variation at the extreme end of the hostility dimension (i.e., an estimated 12.4% of the Dutch population show signs of clinically relevant hostility, given that in the Netherlands an estimated 24.5% of people suffer from a mental disorder (i.e., anxiety, mood, eating, personality and somatoform disorders) in one year [21]. Of these individuals an estimated 51% report moderate levels of anger [22]), we actively recruited participants with increased and clinically relevant levels of hostility from two mental health facilities in the Maastricht area (i.e., METggz and U-Center). Patients with a score above 1.22 on the hostility subscale of the Personality Inventory for DSM-5 (PID-5H) were eligible to enter the study. This cutoff equals 1 SD above the observed mean in a Danish population (a comparable population to the Netherlands) and approximates the mean in a clinical population [23]. Exclusion criteria were age younger than 18 and higher than 60, and illiteracy. Patients [illegible] excluded from participation by clinical judgement in the mental health facility if they showed signs of current psychosis or mania, alcohol or drug abuse/dependency, and acute suicide risk. For exploratory factor analysis, a minimum sample size of $N = 300$ is suggested [24]. In total, we recruited $n = 347$ people from the general population and $n = 30$ patients with clinically relevant levels of hostility. One patient withdrew consent from the study, so the final sample consisted of $N = 376$. Sample characteristics are shown in Table 1. Statistical analyses showed that, compared

really? too much

Add the range and mean and SD for the established cut-off —based on previous studies to understand better this statistical decision

excellent decision based on EFA. By the way, authors have reported several times "exploratory factor analyses" they may use an acronym EFA.

to non-patients, patients were less often female, lower educated, student, and were more often using active psychotropic medication.

Table 1. Sample characteristics.

		Stratum		Statistical dif.
	Total sample (*N* = 376)	Non-clinical (*n* = 347)	Clinical (*n* = 29)	$Z/X^2/t$ *(p)*
Age in years, mean (*SD*)	35.15 (14.72)	34.93 (14.97)	37.72 (11)	1.47 (.141)
Female, *n* (%)	280 (74)	266 (77)	14 (48)	11.34 (.001)
Male, *n* (%)	96 (26)	81 (23)	15 (52)	
Nationality, *n* (%)				2.05 (.563)
Dutch	353 (95)	324 (93)	29 (100)	
Belgian	13 (3)	13 (4)	0 (0)	
German	4 (1)	4 (1)	0 (0)	
Other	6 (2)	6 (2)	0 (0)	
Education, *n* (%)				27.46 (< .001)
Low	91 (24)	89 (26)	2 (7)	
Middle	141 (38)	114 (33)	24 (83)	
High	144 (38)	141 (41)	3 (10)	
Work situation, *n* (%)				26.14 (< .001)
Employed	155 (41)	141 (41)	14 (48)	
Unemployed	42 (11)	36 (10)	6 (21)	
Student	135 (36)	135 (39)	0 (0)	
Social security	41 (11)	32 (9)	3 (10)	
Retired	3 (1)	3 (2)	0 (0)	
Medication use, *n* (%)				
Antidepressant, SSRI	24 (6)	16 (5)	8 (28)	23.64 (< .001)
Antidepressant, SNRI	10 (3)	5 (1)	5 (17)	25.81 (< .001)
Antidepressant, TCA	2 (1)	2 (1)	0 (0)	.17 (.682)
Antidepressant, other	3 (1)	2 (1)	1 (3)	2.79 (.095)
Antipsychotic, atypical	6 (2)	4 (1)	2 (7)	5.62 (.018)
Anxiolytic	10 (3)	5 (1)	5 (17)	25.81 (< .001)
Mood stabilizer	3 (1)	2 (1)	1 (3)	2.79 (.095)
Stimulant	7 (2)	6 (2)	1 (3)	.43 (.511)
Study variables				
STAXI-2T, mean (*SD*)	16.67 (5.21)	16.01 (4.53)	24.76 (6.03)	-7.63 (< .001)
AQH, mean (*SD*)	19.01 (7.25)	18.47 (7.02)	25.45 (7.04)	-5.13 (< .001)
FOA, mean (*SD*)	56.72 (13.12)	55.57 (11.99)	70.52 (17.78)	4.89 (< .001)
PID-5H, mean *(SD)*	.74 (.59)	.66 (.51)	1.65 (.68)	-7.69 (< .001)

SSRI = selective serotonergic reuptake inhibitor; SNRI = selective noradrenergic reuptake inhibitor; TCA = tricyclic antidepressant.

provide here a range of the reported previous reliabilities in every single instrument to be compared with yours

Materials

State Trait Anger Expression Inventory-2

In the 10-item trait anger scale of the State Trait Anger Expression Inventory-2 (STAXI-2T) [25] items (e.g., “I am hot-headed”) are scored on a 4-point Likert scale ranging from one (not at all) to four (very much). Internal consistency, test-retest reliability and concurrent validity are good and adequate construct validity has been demonstrated [25, 26].

Aggression Questionnaire

In the 10-item hostility scale of the Aggression Questionnaire (AQH) [11] items (e.g., “Other people always seem to get the breaks”) are scored on a 5-point Likert scale ranging from one (extremely uncharacteristic of me) to five (extremely characteristic of me). Internal consistency and test-retest reliability are good and adequate/good construct validity has been demonstrated [27, 28].

Forms of Aggression questionnaire

The 40-item Forms of Aggression questionnaire (FOA) [29] comprises a list of harmful behaviors measured across five subscales including physical (e.g., “I hit, kick, or push them”), verbal (e.g., “I say mean things to them”,) property (e.g., “I damage their property”), relational (e.g., “I ruin their friendships with other people”) and passive-rational (e.g., “I criticize their work, even if it is good”) aggression. In the original version people are asked to indicate how often each behavior occurs when angry. To minimalize overlap with affective features of hostility, participants in the current study were asked to indicate how often each behavior occurs *in general* instead. Items are scored on a 5-point Likert scale ranging from one ((almost) never)

better alternative: one or almost never

to five ((almost) always). Good internal consistency and adequate construct, convergent and discriminant validity have been demonstrated [29].

Personality Inventory for DSM-5

In the 10-item hostility scale of the Personality Inventory for DSM-5 (PID-5H) [30] items (e.g., “I snap at people when they do little things that irritate me”) are scored on a 4-point Likert scale ranging from zero (very false or often false) to three (very true or often true). Internal consistency is good and adequate construct and convergent validity have been demonstrated [23, 31-33].

2.3 Procedure

The Ethical Review Committee Psychology and Neuroscience at Maastricht University provided ethical approval to carry out the study (ERCPN- 167_08_05_2016). The study was pre-registered at https://osf.io/gpju6. Some protocol changes were made after the study was preregistered. Specifically, we decided to perform a different analytical approach, we chose to study hostility at trait level (instead of at both state and trait levels) and patients were not screened for instable use of psychotropic medication. The study was performed completely online using Qualtrics software. Beforehand, people were told that the study was about investigating the relationship between thoughts, feelings and behaviors. After participants provided informed consent, an online link to the study’s questionnaires was sent by e-mail. Then, information about demographic variables, use of psychotropic medication were obtained and the PID-5H, STAXI-2T, AQH and FOA were administered. After completion, participants were debriefed and received course credit or participated in a raffle with 347 times €7,50 worth of rewards.

Statistical analysis

IBM SPSS and Amos version 24 were used for statistical analysis. First, descriptive statistics were calculated. Second, Spearman's correlation analysis was run to examine baseline correlations between all multidimensional and unidimensional hostility measures. Then, the hierarchical structure of hostility was examined using the so-called 'Bass-Ackwards' method [34]. Within the context of the examination of personality models, Goldberg (34) provided a method of examining hierarchical structures in models with more than two levels. The approach allows for the examination of various hierarchical levels of specificity, from a broad construct to more fine-grained, lower-level facets that become more specific at each hierarchical level. Factor solutions were identified using Principal Components Analysis. First, an unrotated principal component was extracted, followed by the extraction of successively (i.e., two, three, etc.) more Varimax-rotated principal components. This was then repeated until one of the factors was either too specific to be interpreted (e.g., containing one item) or was no longer interpreta[illegible] containing items that show hardly any content similarity). After each extraction, factor loadings were saved and correlated to compare relationships at each level. The identified principal components were then correlated with the raw scores of the questionnaires.

direct oblimin??

Why Varimax instead of Quartimax? I would explain the reason here

(PCA), you will include this term at least 3 times

Results

Means and standard deviations of study variables are shown in Table 1. All study variables were positively skewed (i.e., the value 0 is outside the +/- 2 * standard error interval of the skewness value). All scores resembled those of other studies using population samples [26, 28, 29, 35-37].

indent this paragraph

First, Spearman's correlations and Cronbach's α's are shown in Table 2. These results suggest that the STAXI-2T, AQH, FOA and PID-5H are significantly positively interrelated. All

correlations were large according to Cohen (38) except for the relationship between the FOA and AQH, that was medium.

Table 2. Spearman's rho correlations between uni- and multidimensional hostility constructs.

	STAXI-2T	AQH	FOA	PID-5H
STAXI-2T	.88			
AQH	.54*	.86		
FOA	.60*	.43*	.93	
PID-5H	.79*	.51*	.65*	.88

Note. *$p < .001$. Cronbach's α is reported in the diagonal.

APA style encourages to include in a last column reliabilities rather than in a diagonal line. FOA is too high by the way (redundacy?)

PCA

Then, to examine the hierarchical structure of hostility a Principal Components Analysis was run using the 'Bass-Ackwards' method. We evaluated multivariate normality and linearity by inspecting Mahalanobis distance. We observed two multivariate outliers who were removed from the analysis. We observed non-normality on eighteen FOA items (i.e., skewness values smaller or larger than three standard errors) [39]. Of these eighteen items fifteen extremely violated the normality assumption even after inverse-transformation (i.e., 1/*x*) and were removed from further analyses to maintain model robustness. The removed items are shown in S1 Table. The first unrotated principal component accounted for 30% of the total variance. The first ten eigenvalues were: 16.16, 3.90, 2.68, 2.12, 1.61, 1.35, 1.24, 1.20, 1.13, and 1.06. Then, successively larger solutions (i.e., two, three, etc.) were examined. Inspection of the 6-principal component solution showed that the last factor consisted of the two

A psychometric work like this should include more figures and tables (loads and a flow-chart of the making decisions about their solutions

items: “I resent being told what to do, even by people in charge” and “I feel annoyed when not given recognition for doing good work”. Thus, the 6-principal component solution was interpreted as not meaningful, resulting in a 5-principal component solution as base of the hierarchical model, accounting for 49% of the variance.

The hierarchical 5-principal component model is shown in Fig 1. Correlations between the component loadings and the original scales are shown in Table 3. Rotated component loadings and item content are shown in S2 Table. All principal components were labeled according to what was most common to all these items. The first component (P1.1) was labeled Hostility and demonstrated significant positive associations to the original hostility scales ranging from r = .66 (AQ-H) to r = .88 (PID-5H). The principal components in the two-factor solution were labeled Hostile Cognition (P2.1) and Aggressive Behavior (P2.2). Hostile Cognition related most strongly to the total scores of the STAXI-2T, AQH and PID-5H. Aggressive Behavior most strongly related to the FOA (r = .92). In the three-component solution the Hostile Cognition component split into an Angry Affect (P3.1) and Hostile Intent (P3.3) component. Angry Affect most strongly related to the STAXI-2T and PID-5H. Hostile Intent most strongly related to the AQH (r = .92). In the four-component solution items from the Aggressive Behavior component split into a Social Aggression (P4.2) and Physical Aggression (P4.4) component. Social Aggression related most strongly to the FOA (r = .87), whereas Physical Aggression related most strongly to the STAXI-2T (r = -.17, p = .001) and FOA (r = .11, p = .029). In the five-component solution content from the Social Aggression component split into a Verbal Aggression (P5.2) and Relational Aggression (P5.4) component. Verbal Aggression (r = .72) and Relational Aggression (r = .35) both most strongly related to the FOA.

Table 3. Spearman’s rho correlations between factor scores and hostility instruments.

	STAXI-2T	AQH	FOA	PID-5H
P1.1	.84***	.66***	.87***	.88***
P2.1	.80***	.77***	.33***	.76***
P2.2	.35***	.14**	.92***	.42***
P3.1	.74***	.18***	.28***	.75***
P3.2	.30***	.18***	.90***	.36***
P3.3	.35***	.92***	.20***	.29***
P4.1	.75***	.18***	.28***	.76***
P4.2	.34***	.18***	.87***	.38***
P4.3	.34***	.92***	.19***	.29***
P4.4	-.17**	.00	.11*	-.08
P5.1	.71***	.16**	.20***	.72***
P5.2	.35***	.13*	.72***	.37***
P5.3	.35***	.93***	.20***	.30***
P5.4	.02	.10	.35***	.05
P5.5	-.12*	.04	.23***	-.03

* significant at $p < .05$; ** significant at $p < .01$; *** significant at $p < .001$.

In the table: editing "*" as superindex to reduce the size of the font

Figure 1. Hierarchical structure of hostility.

Discussion

The present study is, to the best of our knowledge, the first to explore the hierarchical structure of self-reported trait-hostility. We predicted that hostility can be defined as a construct that can be interpreted at different levels of specificity or, in other words, that hostility shows a multidimensional hierarchical structure. We observed that at the highest, most abstract level Hostility is characterized by a low threshold to experience and react harmfully upon angry affect. Findings demonstrate large positive associations between hostility and raw scores on different instruments of hostility. This finding is consistent with different conceptualizations of hostility [2-4, 40-42]. At the second level, hostility splits up into an experiential (Hostile Cognition) and expressive component (Aggressive Behavior), converging with factor analytic studies [9, 10, 12, 13]. Correlations with the original scales show that experiential aspects of hostility are mostly captured by the STAXI-2T, AQH and PID-5H, whereas the expressive aspects are mostly captured by the FOA. At the third level, the experiential factor splits up into an affective (Angry Affect) and cognitive factor (Hostile Intent). This is in line with factor analytic studies that demonstrated a cognitive, affective and behavioral hostility factor [9, 14, 15]. Correlations with the original scales show that the affective component is mostly captured by the STAXI-2T and PID-5H, whereas the cognitive component is mostly captured by the AQ-H. At the fourth level, the behavioral component differentiated in an interpersonal (Social Aggression) and physical (Physical Aggression) component. This largely converges with studies showing a four-factor solution consisting of a cognitive, affective and two behavioral factors [8, 11]. Associations with the original scales show that Social Aggression is mostly captured by the FOA, whereas Physical Aggression was mostly captured by the STAXI-2T and the FOA. Moreover, we demonstrated that the interpersonal component split up into a verbal and relational aggression component. In

short, the present findings show that seemingly diverging factor analytic solutions from previous studies converge into one hierarchically structured model of hostility.

I think it might be pretentious to say this

Similar to other models for which hierarchical structures have shown value (e.g., Big-Five model of personality, intelligence, psychopathology), the current research demonstrates that at the highest, most abstract level 30% of the variance in hostility is explained by one underlying dimension. Already at the second hierarchical level behavioral characteristics are separated from cognitive characteristics, showing that behavior is a clear distinct characteristic within hostility. Moving down another hierarchical layer, interpretational characteristics are separated from affective characteristics. The affective and interpretational components of hostility remain stable facets in the majority (i.e., three out of five) of hierarchical layers, marking their relative stability. At even more specific hierarchical layers, behavioral characteristics of hostility differentiate in three expressive forms of aggressive behavior: physical, verbal and relational aggression. Together, these five facets explain 49% of the variance in hostility items. Overall, these findings show that hostility can be perceived as multifaceted construct in which affective, interpretational and behavioral characteristics are stable components.

Several limitations impact the present findings. First, the present work did not include any discriminant measures. Although the present findings show convergent validity, we cannot draw any definite conclusions on the criterion validity of the present findings. A recommendation for future research is hence to include instruments that show differential relationships to different hostility facets, such as agreeableness, shame proneness, empathy, trust and compassion. Second, the majority of the sample (74%) was female. Given that women exhibit more indirect forms of aggression (e.g., relational, or passive-rational aggression) and men exhibit more direct forms (e.g., physical aggression) [43], the results might differ from a

sample that includes more men. Third, the present work approached hostility from a trait approach. As a result, self-report measures were used. A recommendation for future research is to include measures that capture (state) aspects of hostility on different analytical levels, such as physiologically (e.g., variations in heart-rate variability or skin conductance) or behaviorally (e.g., Competitive Reaction Time Task or Point Subtraction Aggression Paradigm;[44, 45]. Last, in the present study we worked with a selection of instruments that measure hostility constructs. The STAXI-2 and AQ show excellent psychometric properties [25-28] and are extensively used and cited - A Web of Science citation report on 15 November 2019 reveals that the STAXI(-2) and AQ are cited respectively 6160 and 4139 times in scientific articles since 1988. The FOA is less commonly used and cited, but does show good psychometric properties in its original form and closely fits modern definitions of aggressive behavior (i.e., *any* behavior that is intended to cause harm to another person) [1]. Despite this careful questionnaire selection, future research needs to examine whether this hierarchical structure holds when more or other instruments are used.

Another weakness is the lack of predictive measures. Besides, Where's the analyses between clinical and non-clinical samples?

The current finding that hostility is a construct that can be interpreted at different levels of theoretical generality versus specificity comes with several main implications. One implication is that a person can score high on a measure that captures one aspect of hostility, but will not necessarily score high on another. For example, a person with a tendency to be easily angered does not necessarily easily engage in aggressive behavior. Also, a person with the tendency to respond physically aggressive, does not necessarily have the tendency to be verbally aggressive. Hostile affect, cognition and behavior may therefore have different antecedents and consequences, requiring a different approach in clinical context. More importantly, the current study illustrates that the lack of consensus in the current hostility literature is likely the result of

conceptual identity confusion (i.e., jingle and jangle fallacies). This, in turn, leads to reduced measurement precision and a fragmentation of the research field (i.e., two researchers could study the same construct but name their construct differently). Note that hostility is investigated not only in the field of aggression, but also the fields of social psychology, clinical psychology and psychiatry. This underlines the need to be both more critical towards the use of language and to be very precise in choice of measurement instruments in these fields. Hopefully, this study will stimulate joint efforts to move towards the standardized use of hostility and its subcomponents. Moreover, we hope to contribute towards moving the field of aggression research to a more valid and standardized assessment level by further stimulating and ameliorate the accurate and standardized assessment and operationalization of hostility.

References

1. Anderson CA, Bushman BJ. Human aggression. Annu Rev Psychol. 2002;53(1):27-51. Epub 2001/12/26. doi: 10.1146/annurev.psych.53.100901.135231. PubMed PMID: 11752478.

2. Barefoot JC. Developments in the measurement of hostility. In: Friedman HS, editor. Hostility, coping, & health. Washington, DC: American Psychological Association; 1992. p. 13-31.

3. Chaplin JP. Dictionary of psychology. New York, N.Y.: Laurel; 1982.

4. American Psychiatric Association. Diagnostic and Statistical Manual of Mental Disorders. 5th ed. Washington, DC2013.

5. Spielberger CD, Johnson E, Russell S, Crane R, Jacobs G, Worden T. The experience and expression of anger: Construction and validation of an anger and hostility in cardiovascular and behavioral disorders. New York: Hemisphere; 1985.

6. Buss AH. The psychology of aggression. Hoboken, NJ: John Wiley & Sons Inc; 1961.

7. Smith TW. Hostility and health: Current status of a psychosomatic hypothesis. Health Psychol. 1992;11(3):139-50. doi: 10.1037/0278-6133.11.3.139. PubMed PMID: 1992-42983-001.

8. Maier KJ, Goble LA, Neumann SA, Giggey PP, Suarez EC, Waldstein SR. Dimensions across measures of dispositional hostility, expressive style, and depression show some variation by race/ethnicity and gender in young adults. J Soc Clin Psychol. 2009;28(10):1199-225. doi: 10.1521/jscp.2009.28.10.1199. PubMed PMID: 2009-25225-001.

9. Martin R, Watson D, Wan CK. A three-factor model of trait anger: Dimensions of affect, behavior, and cognition. J Pers. 2000;68(5):869-97. doi: 10.1111/1467-6494.00119. PubMed PMID: 2000-00617-003.

10. Musante L, MacDougall JM, Dembroski TM, Costa PT. Potential hostility and dimensions of anger. Health Psychol. 1989;8(3):343-54. doi: 10.1037/0278-6133.8.3.343. PubMed PMID: 1990-00132-001.

11. Buss AH, Perry M. The aggression questionnaire. J Pers Soc Psychol. 1992;63(3):452-9. Epub 1992/09/01. doi: 10.1037/0022-3514.63.3.452. PubMed PMID: 1403624.

12. Buss AH, Durkee A. An inventory for assessing different kinds of hostility. J Consult Psychol. 1957;21(4):343-9. doi: 10.1037/h0046900. PubMed PMID: 1959-01675-001.

13. Fuqua DR, Leonard E, Masters MA, Smith RJ, Campbell JL, Fischer PC. A structural analysis of the State-Trait Anger Expression Inventory. Educ Psychol Meas. 1991;51(2):439-46. doi: 10.1177/0013164491512018. PubMed PMID: 1991-32019-001.

14. Kopper BA, Epperson DL. The experience and expression of anger: Relationships with gender, gender role socialization, depression, and mental health functioning. J Couns Psychol. 1996;43(2):158-65. doi: 10.1037/0022-0167.43.2.158. PubMed PMID: 1996-00434-003.

15. Riley WT, Treiber FA. The validity of multidimensional self-report anger and hostility measures. J Clin Psychol. 1989;45(3):397-404. doi: 10.1002/1097-4679(198905)45:3<397::AID-JCLP2270450308>3.0.CO;2-4. PubMed PMID: 1989-31662-001.

16. Hilgard ER. The trilogy of mind: Cognition, affection, and conation. J Hist Behav Sci. 1980;16(2):107-17.

17. Bacon DR. An evaluation of cluster analytic approaches to initial model specification. Structural Equation Modeling. 2001;8(3):397-429. doi: 10.1207/S15328007SEM0803_4. PubMed PMID: 2001-07608-004.

18. Ashton MC, Lee K, Goldberg LR. A Hierarchical Analysis of 1,710 English Personality-Descriptive Adjectives. J Pers Soc Psychol. 2004;87(5):707-21. doi: 10.1037/0022-3514.87.5.707. PubMed PMID: 2004-20124-011.

19. Lang JWB, Kersting M, Beauducel A. Hierarchies of factor solutions in the intelligence domain: Applying methodology from personality psychology to gain insights into the nature of intelligence. Learning and Individual Differences. 2016;47:37-50. doi: 10.1016/j.lindif.2015.12.003. PubMed PMID: 2016-23853-006.

20. Farmer RF, Seeley JR, Kosty DB, Olino TM, Lewinsohn PM. Hierarchical organization of axis I psychiatric disorder comorbidity through age 30. Comprehensive Psychiatry. 2013;54(5):523-32. doi: 10.1016/j.comppsych.2012.12.007. PubMed PMID: 2014-13520-013.

21. Gustavsson A, Svensson M, Jacobi F, Allgulander C, Alonso J, Beghi E, et al. Cost of disorders of the brain in Europe 2010. Eur Neuropsychopharmacol. 2011;21(10):718-79. doi: 10.1016/j.euroneuro.2011.08.008. PubMed PMID: 2011-23404-006.

22. Posternak MA, Zimmerman M. Anger and aggression in psychiatric outpatients. The Journal of Clinical Psychiatry. 2002;63(8):665-72. doi: 10.4088/JCP.v63n0803. PubMed PMID: 2002-17990-003.

23. Bach B, Maples-Keller JL, Bo S, Simonsen E. The alternative DSM–5 personality disorder traits criterion: A comparative examination of three self-report forms in a Danish population. Personality Disorders: Theory, Research, and Treatment. 2016;7(2):124-35. doi: 10.1037/per0000162

10.1037/per0000162.supp (Supplemental). PubMed PMID: 2015-55074-001.

24. Tabachnick BG, Fidell LS. Using multivariate statistics, 5th ed. Boston, MA: Allyn & Bacon/Pearson Education; 2007.

25. Spielberger CD. State-Trait Anger Expression Inventory-2. Lutz, Florida: Psychological Assessment Resources; 1999.

26. Lievaart M, Franken IHA, Hovens JE. Anger assessment in clinical and nonclinical populations: Further validation of the State–Trait Anger Expression Inventory- 2. J Clin Psychol. 2016;72(3):263-78. doi: 10.1002/jclp.22253. PubMed PMID: 2016-02577-001.

27. McKay MT, Perry JL, Harvey SA. The factorial validity and reliability of three versions of the Aggression Questionnaire using Confirmatory Factor Analysis and Exploratory Structural Equation Modelling. Pers Individ Dif. 2016;90:12-5. doi: 10.1016/j.paid.2015.10.028. PubMed PMID: 2015-57473-009.

28. Hornsveld RH, Muris P, Kraaimaat FW, Meesters C. Psychometric properties of the aggression questionnaire in Dutch violent forensic psychiatric patients and secondary vocational students. Assessment. 2009;16(2):181-92. Epub 2008/10/15. doi: 10.1177/1073191108325894. PubMed PMID: 18852480.

29. Verona E, Sadeh N, Case SM, Reed A, 2nd, Bhattacharjee A. Self-reported use of different forms of aggression in late adolescence and emerging adulthood. Assessment. 2008;15(4):493-510. Epub 2008/06/10. doi: 10.1177/1073191108318250. PubMed PMID: 18539783.

30. Van der Heijden P, Ingenhoven T, Berghuis H, Rossi G. Nederlandstalige bewerking van The Personality Inventory for DSM-5® (PID-5) — Adult, 2011. Amsterdam: Uitgeverij Boom; 2014.

31. Krueger RF, Derringer J, Markon KE, Watson D, Skodol AE. Initial construction of a maladaptive personality trait model and inventory for DSM-5. Psychol Med. 2012;42(9):1879-90. doi: 10.1017/S0033291711002674. PubMed PMID: 2012-23337-010.

32. Thomas KM, Yalch MM, Krueger RF, Wright AGC, Markon KE, Hopwood CJ. The convergent structure of DSM-5 personality trait facets and five-factor model trait domains. Assessment. 2013;20(3):308-11. doi: 10.1177/1073191112457589. PubMed PMID: 2013-22795-005.

33. Watson D, Stasik SM, Ro E, Clark LA. Integrating normal and pathological personality: Relating the DSM-5 trait-dimensional model to general traits of personality. Assessment. 2013;20(3):312-26. doi: 10.1177/1073191113485810. PubMed PMID: 2013-22795-006.

34. Goldberg LR. Doing it all Bass-Ackwards: The development of hierarchical factor structures from the top down. J Res Pers. 2006;40(4):347-58. doi: 10.1016/j.jrp.2006.01.001. PubMed PMID: 2006-07442-001.

35. Ashton MC, de Vries RE, Lee K. Trait variance and response style variance in the scales of the personality inventory for DSM–5 (PID–5). J Pers Assess. 2017;99(2):192-203. doi: 10.1080/00223891.2016.1208210. PubMed PMID: 2017-07812-011.

36. De Fruyt F, De Clercq B, De Bolle M, Wille B, Markon K, Krueger RF. General and Maladaptive Traits in a Five-Factor Framework for DSM-5 in a University Student Sample. Assessment. 2013;20(3):295-307. doi: 10.1177/1073191113475808. PubMed PMID: 23405016.

37. Meesters C, Muris P, Bosma H, Schouten E. Psychometric evaluation of the Dutch version of the Aggression Questionnaire. Behav Res Ther. 1996;34(10):839-43. doi: 10.1016/0005-7967(96)00065-4. PubMed PMID: 1996-01012-009.

38. Cohen J. A power primer. Psychol Bull. 1992;112(1):155-9. doi: 10.1037/0033-2909.112.1.155. PubMed PMID: 1992-37683-001.

39. Curran PJ, West SG, Finch JF. The robustness of test statistics to nonnormality and specification error in confirmatory factor analysis. Psychol Methods. 1996;1(1):16-29. doi: 10.1037/1082-989X.1.1.16. PubMed PMID: 1996-03170-002.

40. Cassiello- Robbins C, Barlow DH. Anger: The unrecognized emotion in emotional disorders. Clin Psychol Sci Pract. 2016;23(1):66-85. doi: 10.1111/cpsp.12139. PubMed PMID: 2016-06124-001.

41. Fernandez E, Johnson SL. Anger in psychological disorders: Prevalence, presentation, etiology and prognostic implications. Clin Psychol Rev. 2016;46:124-35. doi: 10.1016/j.cpr.2016.04.012. PubMed PMID: 2016-24567-001.

42. Vidal-Ribas P, Brotman MA, Valdivieso I, Leibenluft E, Stringaris A. The status of irritability in psychiatry: A conceptual and quantitative review. J Am Acad Child Adolesc Psychiatry. 2016;55(7):556-70. doi: 10.1016/j.jaac.2016.04.014. PubMed PMID: 2016-32356-010.

43. Björkqvist K, Lagerspetz KM, Kaukiainen A. Do girls manipulate and boys fight? Developmental trends in regard to direct and indirect aggression. Aggress Behav.

1992;18(2):117-27. doi: 10.1002/1098-2337(1992)18:2<117::AID-AB2480180205>3.0.CO;2-3. PubMed PMID: 1992-30761-001.

44. Cherek DR, Lane SD, Pietras CJ. Laboratory measures: point subtraction aggression paradigm. Aggression: CRC Press; 2003. p. 231-44.

45. Taylor SP. Aggressive behavior and physiological arousal as a function of provocation and the tendency to inhibit aggression. J Pers. 1967;35(2):297-310. PubMed PMID: 6059850.

Acknowledgements

There are no acknowledgments for the present work.

Supporting information

S1 Table. FOA items that were removed due to extreme normality violation.

S2 Table. Item content and loading for all rotated principal component solutions.

Click here to download Figure Fig 1.tif

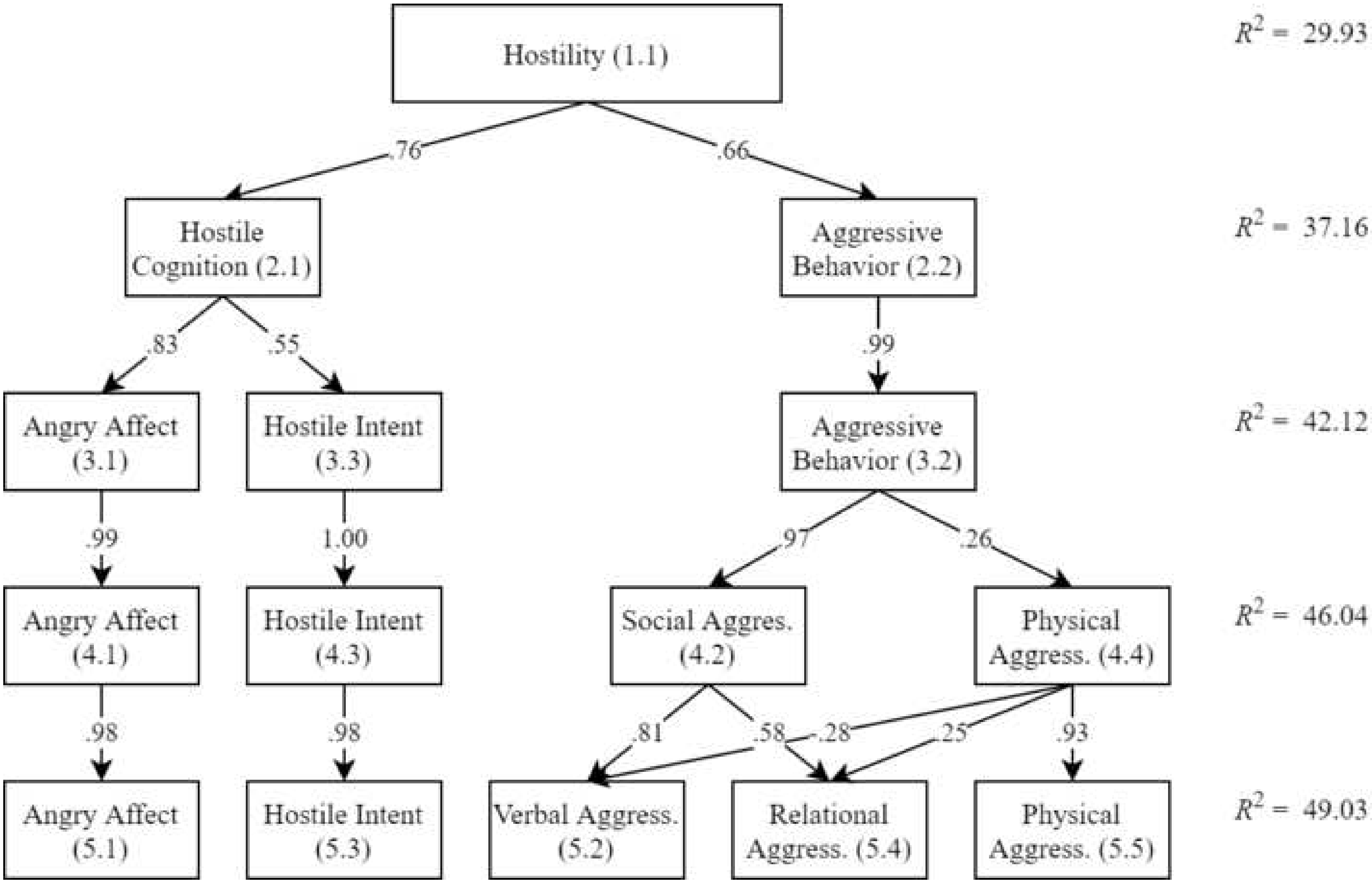

Click here to access/download
Supporting Information
S1_Table.docx

Click here to access/download
Supporting Information
S2_Table.xlsx
