# Supplementary material for: Uncovering the hierarchical structure of self-reported hostility
Source: PLoS One. 2020 Sep 29;15(9):e0239631. doi: 10.1371/journal.pone.0239631 (PMC7523964; doi:10.1371/journal.pone.0239631)
Supplement: Attachment — Submitted filename: Response to Reviewers.docx [file pone.0239631.s007.docx]

Maastricht, 17 August 2020

Dear professor Taubner,

We want to thank the reviewers for the thorough and helpful feedback on the first version of our manuscript, “*Uncovering the hierarchical structure of self-reported hostility*”, and thank you for giving us the opportunity to revise it for PLOS ONE. We appreciate the positive feedback that the reviewers, provided on the paper. Below we reply to each of the issues raised by the reviewers.

**Reply to the editor**

As the reviewers point out there is a need to clarify the gap in hostility research more clearly and a need to justify statistical decisions and sampling in your research.

*Reply*: We thank the editor for providing a concise overview of the reviewers’ comments. We carefully made sure all issues were addressed. See below for our point-by-point reply on these matters.

1. Please ensure that your manuscript meets PLOS ONE's style requirements, including those for file naming. The PLOS ONE style templates can be found at

<https://journals.plos.org/plosone/s/file?id=wjVg/PLOSOne_formatting_sample_main_body.pdf> and

<https://journals.plos.org/plosone/s/file?id=ba62/PLOSOne_formatting_sample_title_authors_affiliations.pdf>

*Reply*: We inspected the template formats in detail and made all the required format changes.

2. We note that you have indicated that data from this study are available upon request. PLOS only allows data to be available upon request if there are legal or ethical restrictions on sharing data publicly. For information on unacceptable data access restrictions, please see http://journals.plos.org/plosone/s/data-availability#loc-unacceptable-data-access-restrictions.

In your revised cover letter, please address the following prompts:

a) If there are ethical or legal restrictions on sharing a de-identified data set, please explain them in detail (e.g., data contain potentially identifying or sensitive patient information) and who has imposed them (e.g., an ethics committee). Please also provide contact information for a data access committee, ethics committee, or other institutional body to which data requests may be sent.

b) If there are no restrictions, please upload the minimal anonymized data set necessary to replicate your study findings as either Supporting Information files or to a stable, public repository and provide us with the relevant URLs, DOIs, or accession numbers. Please see http://www.bmj.com/content/340/bmj.c181.long for guidelines on how to de-identify and prepare clinical data for publication. For a list of acceptable repositories, please see http://journals.plos.org/plosone/s/data-availability#loc-recommended-repositories.

We will update your Data Availability statement on your behalf to reflect the information you provide.

*Reply*: In accordance with publishing standards of PLOS ONE, we have now made the minimal anonymized data set and accompanying syntax available as Supporting Information Files S1_Data.sav and S2_Syntax.sps. This was also added in the manuscript on p. 9 (“*The minimal anonymized data set and accompanying syntax are presented in S1 and S2 Files.”)* and p. 22 (“*S3 File. Minimal anonymized data set.”* and “*S4 File. Syntax accompanying the minimal anonymized data set.*”). S1 Table was renamed to S3 Table and S2 Table to S4 Table to follow order of appearance.

Reviewers' comments:

Reviewer's Responses to Questions

Comments to the Author

1. Is the manuscript technically sound, and do the data support the conclusions?

The manuscript must describe a technically sound piece of scientific research with data that supports the conclusions. Experiments must have been conducted rigorously, with appropriate controls, replication, and sample sizes. The conclusions must be drawn appropriately based on the data presented.

Reviewer #1: Partly

Reviewer #2: Partly

________________________________________

2. Has the statistical analysis been performed appropriately and rigorously?

Reviewer #1: Yes

Reviewer #2: Yes

________________________________________

3. Have the authors made all data underlying the findings in their manuscript fully available?

The PLOS Data policy requires authors to make all data underlying the findings described in their manuscript fully available without restriction, with rare exception (please refer to the Data Availability Statement in the manuscript PDF file). The data should be provided as part of the manuscript or its supporting information or deposited to a public repository. For example, in addition to summary statistics, the data points behind means, medians and variance measures should be available. If there are restrictions on publicly sharing data—e.g. participant privacy or use of data from a third party—those must be specified.

Reviewer #1: No

Reviewer #2: No

________________________________________

4. Is the manuscript presented in an intelligible fashion and written in standard English?

PLOS ONE does not copyedit accepted manuscripts, so the language in submitted articles must be clear, correct, and unambiguous. Any typographical or grammatical errors should be corrected at revision, so please note any specific errors here.

Reviewer #1: Yes

Reviewer #2: Yes

________________________________________

5. Review Comments to the Author

Please use the space provided to explain your answers to the questions above. You may also include additional comments for the author, including concerns about dual publication, research ethics, or publication ethics. (Please upload your review as an attachment if it exceeds 20,000 characters)

**Reply reviewer #1:**

Reviewer #1: The article by van Teffelen and colleagues is clear in its presentation, sound in its statistical approach and addresses an interesting topic in conceptual hostility research.

*Reply*: We much appreciate the positive feedback concerning the presentation and soundness of our statistical approach in addressing hostility research.

Nevertheless, more clarification on statistical decisions is needed in my opinion. First, the authors should address, either in the participants section or at least in the limitations, how the 30 patients might have influenced the results of the analysis. Network models of psychopathology suggest that the strength of the correlation between different symptoms might be directly related to the overall symptom severity of an individual. Consequently, since PCA cannot control for different subgroups (likewise EFA at n = 30) the correlations between the rotated factors and the raw scores might be influenced by different patterns in both groups. While it seems like the logic behind including these 30 patients was to increase the variance of hostile traits in the sample, I could not find a clear statement on why the authors found it necessary to include this subgroup.

*Reply*: We thank the reviewer for bringing the cutting-edge network approach to psychopathology to our attention. Recent work on hostility is indeed in line with findings of network psychopathology models, showing a positive correlation between hostility levels and psychopathological severity. See for example an interesting literature review by Cassiello‐Robbins and Barlow (2016). For this reason, we agree with reviewer #1 that different patterns within subgroups might influence correlations between rotated factors and raw scores. In accordance with Cassiello-Robbins and Barlow’s review we treat hostility as a dimensional (i.e., transdiagnostic) construct. As research indicates that people high in agreeableness (read: low in hostility) have a lower tendency to participate in experiments (Lönnqvist et al., 2007) we indeed wanted to ensure that the sample variance reflected the population distribution. We thus included a statement in the original manuscript on p. 5 indicating: “…*ensuring a representative distribution with enough variation at the extreme end of the hostility dimension*…”. In line with reviewer #1’s valuable suggestions, we have now added two statements to the manuscript, one in the results section on p. 10 (“*Univariate outlier inspection of study variables revealed no bimodality or consistent univariate outliers (following the 3 * interquartile range criterion), suggesting that the patients in the present sample did not form a data-cluster”*) and one in the discussion on p. 16 (“…*30 patients were included in the present sample to ensure enough variation at the extreme end of the hostility dimension. Network models of psychopathology suggest that overall symptom severity is positively related to the strength of correlations between symptom clusters [49]. Recent work shows that hostility is associated with increase psychopathological severity [50].* *Theoretically it could be possible that different patterns in the patient subgroup may impact the present findings, for example by artificially driving up correlations. Nonetheless, absence of bimodality and univariate outliers suggests that hostility levels in the present sample reflect a distribution that might be expected in the population and is in line with the dimensional approach to psychopathology [51].*”).

Second, the correlations of the 4th and 5th principal components with the raw scores seem to require more in-depth discussion. For example, the component dubbed “Physical Aggression” P4.4 is most strongly related to the inverse of the STAXI-2T score. While this might not be unexpected for researchers more familiar with these measures, for me it begets the question how a measure assessing anger is inversely correlated with supposed physical aggression. If this is obvious, please forgive me.

*Reply*: Reviewer #1 is quite accurate in observing the negative association between P4.4 and the STAXI-2T score. In all reviewer #1’s modesty, we agree with reviewer #1 that this association is not obvious. Specifically, this negative association is likely the result of the inverse-transformation (i.e., 1/x) that was performed on some of the more extremely distributed items to obtain normality. These items tend to cluster in P4.4 (at level 4) and P5.5 (at level 5). Some STAXI-2T items include a behavioral description (e.g., “When I get mad I say nasty things”), which explains the negative relationship. We made sure to add the following on p. 12: “*…including many inverse-transformed items….*”. We also added to the discussion on p. 15-16: “*Surprisingly, the Physical Aggression component at level four and five showed a negative association with the STAXI-2T. A likely explanation is that all inversely-transformed items are included in the Physical Aggression component, and that the STAXI-2T includes many items that tap into physical aggressive behavior (e.g., “When I get mad I say nasty things”).*”.

Additionally, components like P5.5 seem to have overall low (albeit significant) associations with all scores which makes me wonder if the gain in explained variance is justified. An addition to table 3 showing the increase in R² at each step would be helpful. Thank you for your work and excuse my comments if inappropriate.

*Reply*: We agree with reviewer #1 that adding a fifth level to the hierarchical model requires solid justification. However, Goldberg’s bass-ackwards method (see statistical analysis section) entails the extraction of factors until one of the factors is either too specific to be interpreted (e.g., containing one item) or is no longer interpretable (e.g., by containing items that show hardly any content similarity). The increase in *R*^2^ from level 4 to level 5 is 2.99 (as is shown in Fig 1). We belief that this increase in *R*^2^ is still substantial enough (i.e., the increase from 1 component to 2 components is 7.19) not to deviate from Goldberg’s method, as P5.5 is still interpretable and not too specific.

We also added to Table 3’s note: “*R2-values of each level are respectively: 29.93, 37.16, 42.12, 46.04, and 49.03.*”.

**Reply reviewer #2:**

Reviewer #2: First of all, I very much appreciate the effort of the authors to show some light on a construct like hostility where there seems to be no clear consensus yet.

*Reply*: We thank reviewer #2 for the positive feedback concerning our efforts to shed light on a construct that even social-psychologist Bandura referred to as a ‘semantic Jungle’ (Bandura, 1973).

The AHA model (aggression, hostility, and anger) is the most accepted model and the one that is most clearly understood by assigning each part of the behavior to a specific dimension. Thus, anger is emotional, aggression is the behavioral and hostility is cognitive to the respectively dimensions of the AHA. Therefore, I do not finish seeing in what this work contributes to show a hierarchical structure on hostility as a whole instead of as a part of the AHA. I would like to see clearly what is the gap of a specific literature review that this work tries to fill or cover. They did not convince me yet.

*Reply*: We are aware that there are different opinions in the field on how human antagonistic behavior and its’ related cognitive affective experiences are operationalized. We agree with Reviewer #2 that the AHA model is an influential model in explaining self-reported hostility. We therefore now specifically refer to the AHA, or ABC model in lines 56-57 of the revised version of this paper. Indeed, in line with the AHA model, some psychometric studies find support for a three-factor model. Other studies however, find support for a two-factor model with no differentiation between cognitive and affective factors, or a ‘more-than-three’ factor model with further differentiation between behavioral factors. In line with the latter, even Spielberger, one of the profound advocates of the AHA model differentiates between emotions that are expressed as behavior, or ‘internally’ expressed (Spielberger et al., 1985). However, the validity of a construct is determined by the precision of the instruments that measure it. Close inspection of items that have the measurement-pretention of capturing emotion, cognition and behavior in fact reveals that many items cross-capture hostility elements. Examples that capture both behavior and emotion are: `How often one shows certain aggressive behaviors *when angry*` (Reactive Proactive Questionnaire, Raine et al., 2006), or “I have become so mad that I have broken things” (Buss-Perry Aggression Questionnaire, Buss & Perry, 1992), or “When I get mad I say nasty things`` (State-Trait Anger Expression Inventory-2, Spielberger, 1999).

This illustrates that the validity of these items, already at face level, is questionable. In sum, at this point, the optimal factor solution of hostility is debatable. It is not clear how different solutions relate to each other and how central hostility facets are to the broad hostility domain. To stress this, we now added the following text to p. 4: “*Theoretically the ABC- or AHA-model has been influential. However, empirical evidence shows that the optimal factor structure of hostility is debatable. Moreover, it is unclear how different homogenous facets relate to each other and how central they are to the broad-hostility domain. Lack of consensus leads to measurement imprecision. Close inspection of item-content in widely adapted measures of hostility facets for example shows that items often cross-capture hostility facets. For example, how often one shows certain aggressive behaviors when angry (Reactive Proactive Questionnaire) [21], or “I have become so mad that I have broken things” (Buss-Perry Aggression Questionnaire) [9], or “When I get mad I say nasty things” (State-Trait Anger Expression Inventory-2) [6]*”

To compare the tradition of psychometric studies of personality and intelligence with hostility is not pretty accurate and it is not adequate to include it as a premise to conduct this study. It is just an opinion and not a suggestion.

*Reply*: We thank the reviewer for pointing out that using personality or intelligence as examples can be perceived as inadequate. As our only goal in addressing personality and intelligence was exemplification, we now provide more adequate examples on p. 5: “*For example, within the construct of narcissism it has been shown that seemingly diverging results of factor analytic studies (i.e., showing different ‘optimal’ factor solutions) converge into a five-layered hierarchical model in which lower-order facets become more and more specific with each hierarchical layer [23]. Other examples of presumed diverging models that converge into a multi-layered hierarchical model have been reported for agreeableness [24], impulsivity [25], emotion expression [26], and avoidance behavior [27].*”

Results should provide more tables and flow decisions according to APA style for psychometric studies. For example, I needed to read some psychometric-key elements in the MS:

Accuracy: The closeness of a measurement, calculation, or specification to the correct value. Contrasted with precision (the degree of refinement of the measurement, etc.)

Appropriate: Suitable or proper in the circumstances

Validity: Well-founded on fact, or established on sound principles, and thoroughly applicable to the case or circumstances; soundness and strength (of argument, proof, authority, etc.)

*Reply*: We agree with reviewer #2 that self-report instruments should possess sound psychometric properties. Accuracy of an instrument can be investigated by examining criterion validity; precision can be investigated by investigating reliability; appropriateness concerns the selection of facets after accuracy has been obtained; and validity, can be assessed by investigating different forms of validity (e.g., construct validity, discriminant validity).

The study was accurate but I found some lack of appropriate (cultural biases in the sample, clinic and non-clinic participants) and validity (especially, predictive validity, supplemental documentation with a final scale using or suggesting items, divergent instruments, etc..)

*Reply*: The goal of the present study was to map the organization of hostility based on existing hostility measures. The goal of the present work was not to develop a new measure. With regard to the appropriateness of using a mixed community-clinical sample, see our reply to reviewer #1, where we argue that it could be possible that different patterns in the patient subgroup may impact the present findings, for example by artificially driving up correlations.. We agree with reviewer #2 that cultural bias impacts the present work. The results may for example not generalize to an Italian sample. We made sure to address this in the limitation section on p. 16: “ *Fourth, all self-reports were administered in Dutch. Consequently, the results of present work may be culturally bias and may not generalize to non-Dutch cultures.*” We also agree with reviewer #2 that validity is highly important. The present work concerns an investigation of construct validity (i.e., structure) and some degree of discriminant validity (i.e., convergent validity). However, the present work is limited in divergent validity. See, for example, lines 295-297: “*First, the present work did not include any predictive measures. Although the present findings show convergent validity, we cannot draw any definite conclusions on the criterion validity of the present findings.*” In line with reviewer #2’s suggestions, we now highlight items that may be less appropriate, as they cross-capture hostility facets. We refer to this on p. 18: “*This includes, for instance, the use of items that cross-capture hostility facets, while they pretend to measure only one facet (see S2 Table for examples of items that capture multiple facets).*”

I need that authors demonstrate why this study is really necessary and what gaps they tried to fill in the measurement of the hostility.

*Reply*: We thank reviewer #2 for his thorough appraisal of our work. For more detailed replies on the theoretical background and necessity of the present work, see our previous replies. In general, we believe that the present work contributes to the organization of hostility facets in a literature that lacks consensus. See, for example literature reviews by Cassiello‐Robbins and Barlow (2016); Fernandez and Johnson (2016); Ramirez and Andreu (2006); Vidal-Ribas, Brotman, Valdivieso, Leibenluft, and Stringaris (2016).

We hope this will fuel the development of standardized and more precise instrument that capture hostility facets.

**Reply to in-text comments of Reviewer #2**

“This affirmation is not based on specific AHA (aggression, hostility, and anger) literature. Aggression is the behavioral, hostility the cognitive, and anger is the emotional component of the triad. Often???” (p. 2), and “Provide some examples of such affirmation” (p. 3).

*Reply*: We now provide examples on p. 3: “*For example, one may refer to hostility as the ‘cognitive component’ [2, 3], while others refer to hostility as the interrelated elements of cynical beliefs, angry feelings and aggressive responding [4]. Some have referred to anger as the ‘affective component’ [5, 6], while others refer to anger as a combination of cognitive (i.e., biased information processing) and affective factors [7]. There are researchers who refer to anger as ‘irritability’ [8]. Also, the behavioral component of hostility is often restricted to observable aggressive behavior [1], while others refer to aggression as the sum of physical/verbal aggressive behavior, attitudinal hostile beliefs and angry responsiveness [9].*”

“Please, including here a specific citation for this affirmation. This premise requires a further explanation of why they have considered to include these (yellow tracked) expressions. I'd like to see at least a citation of this previous findings” (p. 4)

*Reply*: We thank the reviewer for pointing out this grammatical inconsistency. As we refer to all the combined studies that are mentioned in this paragraph, we now state: “*Taken together, the findings of methodologically heterogeneous factor analytic studies hardly converge in terms of number of produced factors and factor content.*”

“g, third stratum of CHC cognitive ability model should be represented italicized. I could not see the parallelism between hostility and personality or intelligence. The complexity of personality or intelligence are above of the hostility one. Are you considering here a psychometric approach for hostility?” (p. 4).

*Reply*: See our earlier reply. We modified our examples of g and personality to more specific personality constructs.

“really? too much” (p. 5).

*Reply*: We carefully reinspected the study by Gustavson et al. that reported 1-year prevalence rates of mental disorders in the Netherlands. Together, 4070832 people out of 16574989 suffered from a mental health disorder excluding psychosis and addiction. This equals 24.5%.

“Add the range and mean and SD for the established cut-off —based on previous studies to understand better this statistical decision” (p. 5)

*Reply*: This information is provided on p. 6: “This cutoff equals 1 SD above the observed mean in a Danish population (a comparable population to the Netherlands) and approximates the mean in a clinical population [23].”

“excellent decision based on EFA. By the way, authors have reported several times "exploratory factor analyses" they may use an acronym EFA.” (p. 5) “(PCA), you will include this term at least 3 times” (p. 9)

*Reply*: We thank the reviewer for this considerate recommendation. We adjusted this in the manuscript accordingly.

“provide here a range of the reported previous reliabilities in every single instrument to be compared with yours”

*Reply*: For comparability we now provide Cronbach’s alpha ranges for each instrument.

“direct oblimin?? Why Varimax instead of Quartimax? I would explain the reason here.” (p. 9)

*Reply*: Following the reviewer’s suggestion we have added on p. 10: “*Varimax rotation was opted following Goldberg (2006) because of optimal parsimony and to encourage factor markers that are maximally unrelated to each other.*”

“APA style encourages to include in a last column reliabilities rather than in a diagonal line. FOA is too high by the way (redundancy?)” (p. 10)

*Reply*: In line with the reviewer’s suggestion we have added a column with reliabilities. We have maintained the FOA reliability in the table to be consistent. We now also added the internal consistencies that were previously reported in the literature on p. 8. These values are in line with the alpha values we found in our dataset.

“A psychometric work like this should include more figures and tables (loads and a flow-chart of the making decisions about their solutions” (p. 11)

*Reply*: In line with the reviewer’s suggestion, factor loadings are presented in the original version of the manuscript in S2 Table (now S4 Table). Unlike traditional factor analysis, Goldberg’s bass-ackwards approach entails to continue drawing factors until a factor is either too specific to be interpreted (e.g., containing one item) or is no longer interpretable (e.g., by containing items that show hardly any content similarity). To provide some extra information, we now added on p. 11: “*Factor loadings are presented in S4 Table. S5 Fig shows the decision process for principal component extraction. First, one unrotated principal component was extracted, followed by the extraction of successively (i.e., two, three, etc.) more Varimax rotated principal components. This was then repeated until one of the factors was either too specific to be interpreted (e.g., containing one item) or was no longer interpretable (e.g., by containing items that show hardly any content similarity).*”.

“I think it might be pretentious to say this” (p. 14).

*Reply*: We have adjusted this according to your earlier suggestion, in line with the introduction. P. 15: “*…narcissism, agreeableness, impulsivity, avoidance behavior, emotional expression…*”.

Another weakness is the lack of predictive measures. Besides, Where's the analyses between clinical and non-clinical samples?

*Reply*: We agree with the reviewer that a lack of predictive measures is a limitation of the present study. We have addressed this as the first limitation in the original manuscript. However, we now refer to ‘discriminant measures’ as ‘predictive measures’ on p. 16: “*First, the present work did not include any predictive measures. Although the present findings show convergent validity, we cannot draw any definite conclusions on the criterion validity of the present findings.*” The analysis between was addressed in our comment to Reviewer #1; we now argue in the limitations section that different patterns in patients may artificially drive up correlations. We agree with Reviewer #1 that the sample for running an EFA on *n* = 30 patients is too small.

________________________________________

6. PLOS authors have the option to publish the peer review history of their article (what does this mean?). If published, this will include your full peer review and any attached files.

If you choose “no”, your identity will remain anonymous, but your review may still be made public. Do you want your identity to be public for this peer review? For information about this choice, including consent withdrawal, please see our Privacy Policy.

Reviewer #1: No

Reviewer #2: Yes: Jose M Mestre ORCID: https://orcid.org/0000-0002-6822-4970

While revising your submission, please upload your figure files to the Preflight Analysis and Conversion Engine (PACE) digital diagnostic tool, https://pacev2.apexcovantage.com/. PACE helps ensure that figures meet PLOS requirements. To use PACE, you must first register as a user. Registration is free. Then, login and navigate to the UPLOAD tab, where you will find detailed instructions on how to use the tool. If you encounter any issues or have any questions when using PACE, please email PLOS at figures@plos.org. Please note that Supporting Information files do not need this step.

*Reply*: Figures were uploaded and adjusted.

We again want to thank you and the reviewers for all the helpful comments, which we believe have strengthened our manuscript. We hope we were able to address all comments raised by the reviewers to your satisfaction, and that you consider this revised version of the manuscript to be improved and suitable for publication in *PLOS ONE*.

Sincerely, also on behalf of the co-authors,

Martijn van Teffelen

**References**

Bandura, A. (1973). Aggression: A social learning analysis. In (pp. 2): prentice-hall.

Buss, A. H., & Perry, M. (1992). The aggression questionnaire. *Journal of Personality and Social Psychology, 63*(3), 452-459. doi:10.1037/0022-3514.63.3.452

Cassiello‐Robbins, C., & Barlow, D. H. (2016). Anger: The unrecognized emotion in emotional disorders. *Clinical Psychology: Science and Practice, 23*(1), 66-85. doi:10.1111/cpsp.12139

Fernandez, E., & Johnson, S. L. (2016). Anger in psychological disorders: Prevalence, presentation, etiology and prognostic implications. *Clinical Psychology Review, 46*, 124-135. doi:10.1016/j.cpr.2016.04.012

Lönnqvist, J.-E., Paunonen, S., Verkasalo, M., Leikas, S., Tuulio-Henriksson, A., & Lönnqvist, J. (2007). Personality characteristics of research volunteers. *Eur J Pers, 21*(8), 1017-1030. doi:10.1002/per.655

Raine, A., Dodge, K., Loeber, R., Gatzke-Kopp, L., Lynam, D., Reynolds, C., . . . Liu, J. (2006). The reactive-proactive aggression questionnaire: Differential correlates of reactive and proactive aggression in adolescent boys. *Aggressive Behavior, 32*(2), 159-171. doi:10.1002/ab.20115

Ramirez, J. M., & Andreu, J. M. (2006). Aggression, and some related psychological constructs (anger, hostility, and impulsivity); some comments from a research project. *Neuroscience and biobehavioral reviews, 30*(3), 276-291. doi:10.1016/j.neubiorev.2005.04.015

Spielberger, C. D. (1999). *State-Trait Anger Expression Inventory-2*. Lutz, Florida: Psychological Assessment Resources.

Spielberger, C. D., Johnson, E., Russell, S., Crane, R., Jacobs, G., & Worden, T. (1985). The experience and expression of anger: Construction and validation of an anger and hostility in cardiovascular and behavioral disorders. In: New York: Hemisphere.

Vidal-Ribas, P., Brotman, M. A., Valdivieso, I., Leibenluft, E., & Stringaris, A. (2016). The status of irritability in psychiatry: A conceptual and quantitative review. *Journal of the American Academy of Child and Adolescent Psychiatry, 55*(7), 556-570. doi:10.1016/j.jaac.2016.04.014
